# LIN28B-AS1-IGF2BP1 binding promotes hepatocellular carcinoma cell progression

**DOI:** 10.1038/s41419-020-02967-z

**Published:** 2020-09-11

**Authors:** Jian Zhang, Kewei Hu, Yong-qiang Yang, Yin Wang, Yu-fan Zheng, Yong Jin, Ping Li, Long Cheng

**Affiliations:** 1 Department of Interventional Radiology, the Second Affiliated Hospital of Soochow University, Suzhou, China; 2 Department of Gastroenterology, the Second Affiliated Hospital of Soochow University, Soochow University, Suzhou, China; 3 Department of Radiotherapy and Oncology, the Second Affiliated Hospital of Soochow University, Suzhou, China; 4 Jiangsu Key Laboratory of Neuropsychiatric Diseases and Institute of Neuroscience, Soochow University, Suzhou, China; 5 Department of Radiotherapy and Oncology, Affiliated Kunshan Hospital of Jiangsu University, Kunshan, China

**Keywords:** Oncogenes, Targeted therapies

## Abstract

IGF2BP1 overexpression promotes hepatocellular carcinoma (HCC) progression. Long non-coding RNA LIN28B-AS1 directly binds to IGF2BP1. In the present study, LIN28B-AS1 and IGF2BP1 expression and their potential functions in HCC cells were tested. Genetic strategies were applied to interfere their expression, and cell survival, proliferation and apoptosis were analyzed. We show that LIN28B-AS1 is expressed in established/primary human HCC cells and HCC tissues. RNA-immunoprecipitation (RIP) and RNA pull-down results confirmed that LIN28B-AS1 directly associated with IGF2BP1 protein in HCC cells. LIN28B-AS1 silencing (by targeted siRNAs) or knockout (KO, by CRISPR-Cas9 method) depleted IGF2BP1-dependent mRNAs (*IGF2*, *Gli1*, and *Myc*), inhibiting HCC cell growth, proliferation, migration, and invasion. Conversely, ectopic overexpression of LIN28B-AS1 upregulated IGF2BP1-dependent mRNAs and promoted HCC cell progression in vitro. Importantly, ectopic IGF2BP1 overexpression failed to rescue LIN28B-AS1-KO HepG2 cells. LIN28B-AS1 siRNA and overexpression were ineffective in IGF2BP1-KO HepG2 cells. In vivo, LIN28B-AS1 KO-HepG2 xenograft tumors grew significantly slower than the control tumors in the nude mice. Taken together, we conclude that LIN28B-AS1 associates with IGF2BP1 to promote human HCC cell progression in vitro and in vivo.

## Introduction

Hepatocellular carcinoma (HCC) is a primary cause of cancer-associated human mortalities. The overall 5-year survival of HCC is far from satisfactory^1–3^. Due to the lack of effective treatments, patients with advanced, recurrent and/or metastatic HCCs often have extremely poor prognosis^1–3^. Furthermore, HCC incidence has been rising in recent years^1–3^. Therefore, novel and more efficient anti-HCC strategies are needed^1–3^.

Insulin-like growth factor 2 (IGF2) mRNA-binding protein 1 (IGF2BP1) is a primary member of the conserved IGF2BP RNA-binding family proteins^4^. IGF2BP1-dependent mRNAs encode oncogenic proteins, essential for neoplastic transformation and cancer cell progression^4^. IGF2BP1 binds to *IGF2 mRNA*, which is required for its stabilization and translation^4^. IGF2BP1 is also essential for the mRNA stabilization and translation of several other oncogenic genes, including *glioma-associated oncogene homolog 1* (*Gli1*), *Myc* and *CD44*^5^. IGF2BP1 is overexpressed in human HCC, which is associated with cancer progression and poor prognosis^6–9^. Targeting IGF2BP1 can efficiently inhibit human HCC cells^6–9^.

Long non-coding RNAs (LncRNAs) are over 200-nt long non-coding RNA (ncRNA) molecules^10–12^. LncRNAs can function as molecular signals, decoys, guides, scaffolds, or enhancers to regulate gene transcription, expression, and functions^10–12^. Besides, LncRNAs are actively involved in regulating a number of key cellular behaviors, including genomic imprinting, cell cycle control, cell differentiation, pluripotency maintenance, and development^10–12^.

LncRNAs are dysregulated in human HCC, associated with the clinico-pathological features of human HCC^10–13^. For example, Wang et al. reported that upregulated LncRNA UCA1 promoted HCC progression via inhibition of microRNA-216b^14^. The study by Yuan et al. showed that LncRNA DANCR enhanced HCC’s stemness features by directly binding and inhibiting *CTNNB1*^15^. Quagliata and colleagues have shown that LncRNA HOTTIP/HOXA13 expression in HCC patients is associated with disease progression and predicts clinical outcome^16^. A very recent study by Wang et al. has discovered a novel cancer-testis specific LncRNA, namely LIN28B-AS1^17^. It is expressed in lung adenocarcinoma and interacts directly with the IGF2BP1 protein^17^. The results of the present study will show that LIN28B-AS1 is vital for IGF2BP1’s functions, promoting HCC cell progression in vitro and in vivo.

## Methods

### Chemicals, reagents, and antibodies

Cell culture reagents were provided by Hyclone Co. (Logan, UT). Puromycin was purchased from Sigma-Aldrich (St. Louis, MO). Antibodies for IGF2BP1 (#8482), Gli1 (#2643), c-Myc (#18583), and β-tubulin (#15115) were purchased from Cell Signaling Tech (Beverly, MA). The anti-IGF2 antibody (sc-515805) was obtained from Santa Cruz Biotech (Santa Cruz, CA). All the primers and sequences were designed and provided by Shanghai Genechem Co. (Shanghai, China).

### Culture of established cells

Cultures of the established HepG2 and Huh-7 HCC cell lines as well as HL-7702 human hepatocytes were described in previous studies^18,19^.

### Culture of primary human cells

As described^19^, two different human HCC tissues were digested by collagenase I (Sigma). Blood vessel cells, fibroblasts, immune cells, and other non-cancerous cells were abandoned. Primary HCC cells were cultured in the described medium^19^. Two different sets of primary HCC cells were named as “HCC1” and “HCC2”. These primary HCC cells are proliferative. The cell doubling time is about 2.5 days for HCC1 cells and 4 days for HCC2 cells. Human primary adult hepatocytes, purchased from the Cell Bank of Fudan University (Shanghai, China), were derived from the liver of a partial hepatectomy patient. Human hepatocytes were cultured in primary cell culture medium^20^. The written-informed consent was obtained from each participant. Experiments and protocols requiring human tissues/cells were approved by the Ethics Board of Soochow University, according to Declaration of Helsinki. Cells in the present study were routinely subjected to mycoplasma and microbial contamination examination for every 3–4 months. Authentication by STR profiling, population doubling time, and cell morphology were checked as well to confirm the genotype.

### Human tissues

A total of seven (7) written-informed consent primary HCC patients (“P1–P7”, 45–69 years old), administrated at the Affiliated Hospitals of Soochow University, were enrolled. Tumor tissues and the surrounding normal liver tissues were separated carefully by the operating microscopes. The tissues were minced and homogenized by the tissue lysis buffer (Beyotime Biotechnology, Wuxi, China). Written-informed consent was provided by each participant.

### RNA extraction and qPCR

RNA extraction and quantitative real time-PCR assay (qPCR, using the ABI Prism 7500 Fast Real-Time PCR system) were performed as described^21^. The melt curve analysis was applied to calculate product melting temperature. *GAPDH* was tested as the reference gene, using the 2^−∆∆Ct^ method to quantify target RNAs. The mRNA primers for *IGF2*, *Myc*, and *Gli1* as well as *U6* were provided by Dr. Wang^21^. The primers of LIN28B-AS1 (based on ref. ^17^) were synthesized by Genechem (Shanghai, China). LIN28B-AS1 expression was normalized to U6.

### RNA-immunoprecipitation (RIP)

Briefly, following the applied treatment, cells were collected by trypsinization, washed, and incubated with 0.3% formaldehyde and glycine (0.125 M), and cell pellets were re-suspended in the RIP buffer as previously described^22^. Lysates were then incubated with the anti-IGF2BP1 antibody (Santa Cruz Biotech). IGF2BP1-bound pellets were washed, re-suspended and incubated with three times in cold PBS, and re-suspended and incubated with the proteinase K-containing buffer containing. IGF2BP1-bound RNA was isolated. LIN28B-AS1 expression was tested by qPCR.

### RNA pull-down

Biotin-labeled full-length LIN28B-AS1 (see ref. ^17^) was transcribed using the described protocol^21^, isolated with the RNeasy Mini kit (Invitrogen). Biotinylated LIN28B-AS1 was dissolved in RNA structure buffer and folded, put on ice immediately, and then transferred to room temperature. For each treatment, 500 μg cleared nuclei lysates of cultured cells were mixed with folded LIN28B-AS1 and Dynabeads MyOne Streptavidin C1 magnetic beads (“Beads”, provided by Dr. Wang^21^). Beads were washed, and the retrieved proteins were tested by Western blotting.

### LIN28B-AS1 siRNA

Cells were seeded into the six-well tissue culture plates (1 × 10^5^ cells per well). Two different small interfering RNAs (siRNAs) against non-overlapping sequence of LIN28B-AS1 were synthesized by Genechem (Shanghai, China), with the sequence “S1”, 5′-*UUCAAGGGUGGCUGAAACAACAAAAAUGU*-3′ and “S2”, 5′-*UUCAAGGGUGGCUGAAACAACAAA*-3′. LIN28B-AS1 siRNA (0.5 μM) was transfected by Lipofectamine 2000 reagent (Invitrogen) for 24 h, repeated for second round for another 24 h. LIN28B-AS1 knockdown was confirmed by qPCR. Control cells were transfected with the scramble non-sense control siRNA (“siR-C”, Genechem, Shanghai, China).

### LIN28B-AS1 knockout (KO)

Cells were seeded into the six-well tissue culture plates (1 × 10^5^ cells per well) with 60% confluence. LIN28B-AS1 sgRNA (Target DNA sequence, 5′-*GGATGCCCTGGACATCATTCCG*-3′) was annealed into the BbsI-linearized pSpCas9(BB)-2A-GFP (PX458) plasmid (Addgene, Cambridge, MA). Cells were transfected with the construct via Lipofectamine 2000. FACS sorting of the GFP-positive cells were performed, and selected monoclonal cells were cultured for another 10–12 days. LIN28B-AS1 genotyping was performed. The stable cells (two lines) with LIN28B-AS1 depletion were then established.

### LIN28B-AS1 overexpression

The full-length pre-LIN28B-AS1 (see listed sequence^17^) was amplified from HepG2 cells using the previously described primers^5^. The pre-LIN28B-AS1 was inserted to the pLenti6-puro-GFP vector (Invitrogen). The pLenti6-puro-GFP-LIN28B-AS1 expression vector (“LV-LIN28B-AS1”) was transfected to HepG2 cells. Cells were then subjected to puromycin (1.0 μg/mL) selection for another 4–6 passages. Two stable HepG2 cell lines with LV-LIN28B-AS1 construct (“L1/L2”) were established. LIN28B-AS1 overexpression in stable cells was confirmed by qPCR. Control cells were transfected with pLenti6-puro-GFP vector control (“LV-C”).

### Cell viability assay

Cells were initially seeded into the 96-well tissue culture plates (3 × 10^3^ cells per well). MTT (Sigma) assay was performed to test the cell viability. MTT optical density (OD) at 550 nm was recorded.

### BrdU incorporation

Cells were seeded into the 24-well tissue culture plates (2 × 10^5^ cells per well) at 60% confluence. Cell proliferation was detected using a BrdU incorporation ELISA kit (Cell Signaling, Shanghai, China) after 48 h. Cells were incubated with BrdU (10 μM), with BrdU absorbance value tested at 450 nm.

### EdU assay

As previously described^21^ the 5-ethynyl-20-deoxyuridine (EdU) Apollo-488 Kit (Ribo-Bio, Guangzhou, China) was utilized for the quantification of cell proliferation. Following the applied genetic treatments, HCC cells were cultured for 48 h and stained with EdU (10 μM, 2 h at room temperature). Cell nuclei were co-stained with DAPI for 10 min, visualized under a fluorescent microscope (Leica).

### In vitro cell migration and invasion assays

Corning chambers (12 μm pore, Corning, New York, NY), pre-coated with or without Matrigel (0.5 mg/mL, BD Biosciences, Shanghai, China), were utilized. HCC cells with applied genetic treatments (1 × 10^5^ cells of each treatment), starved overnight, were added to the upper chamber, with lower chamber filled with completed medium (with 10% FBS). After 16 h, HCC cells invaded to the lower surface of the chamber were fixed, stained, and counted. Mitomycin (1.5 μg/mL, Sigma) was always added to exclude the influence of cell proliferation^21^.

### TUNEL assay of cell apoptosis

HCC cells with the applied genetic treatments were initially seeded into six-well tissue-culture plates (at 1 × 10^5^ cells per well), and cultured for 48 h. A TUNEL Kit (Invitrogen Thermo-Fisher, Shanghai, China) was utilized. TUNEL and DAPI dyes were added to HCC cells, visualized under a fluorescent microscope.

### Annexin V FACS

Cells with the applied genetic modifications were stained with Annexin V-FITC (15 μg/mL) and PI (15 μg/mL) (Biyuntian, Wuxi, China), and detected via fluorescence-activated cell sorting (FACS) on a FACSCalibur machine (BD Biosciences). Annexin V^+/+^ cells were labeled as the apoptotic cells, and its ratio was recorded.

### JC-1 assay of mitochondrial depolarization

Apoptotic cells will often undergo mitochondrial depolarization (“∆Ψ”), and JC-1 dye shall aggregate in mitochondria to form green monomers^23^. HCC cells with the applied genetic treatments were incubated with JC-1 (5 μg/mL) for 15 min under the dark. JC-1 green fluorescence intensity, at 550 nm, was examined by a fluorescence spectrofluorometer (Titertek Fluoroscan, Germany). The representative JC-1 images, intergrading green and red fluorescence images, were presented as well.

### Western blotting

Cells or tumor tissues were harvested via RIPA lysis buffer (Biyuntian, Nanjing, China). Total protein was quantified, mixed with 5× sample buffer, and boiled at 95 °C for 5 min. Aliquots of 40 μg lysate proteins per sample were separated by SDS–PAGE gels, and transferred to the PVDF blots, followed by detection with the indicated primary and secondary antibodies. An enhanced chemiluminescence (ECL) detection kit (Amersham, Buckinghamshire, UK) was utilized to visualize the targeted protein bands. Band intensity was quantified by ImageJ software (NIH).

### IGF2BP1 KO

HepG2 cells were seeded onto six-well tissue culture plates (1 × 10^5^ cells per well). CRISPR/Cas9-IGF2BP1-KO construct (with sgRNA 5′-*GAGCACAAGATCTCCTACAG*-3′, from Dr. Liu^24^) was transfected to HepG2 cells. FACS sorting of the GFP-positive cells was performed, and cells further cultured for another 10–12 days. Monoclonal cells were then subjected to genotyping of depleted region of IGF2BP1. Two lines of stable HepG2 cells with complete IGF2BP1 KO were established, with IGF2BP1 KO confirmed by Western blotting.

### IGF2BP1 overexpression

The recombinant adenovirus encoding the human IGF2BP1 pSUPER-puro construct was provided by Dr. Liu^24^, added to HepG2 cells. Infection was allowed to proceed for 48 h. Expression of IGF2BP1 in the resulting cells was tested by Western blotting.

### In vivo tumor growth

As reported^19^, HepG2 cells were injected subcutaneously (s.c.) to the right flanks of female nude mice (6–7 weeks old, 18–19 g). When tumors reached close to 100 mm^3^, mice were randomized into four groups (10 mice per group). Tumor volumes and mice body weights were monitored every 7 days. Tumor volumes were calculated via the formula: (mm^3^) = (the shortest diameter^2^ × the longest diameter)/2. For recording mice body weight, the estimated tumor weight (tumor volume × 1 mg/mm^3^) was always subtracted from total mice weight. All injections were performed via the described anesthesia method^25^. All animal studies were performed according to the standards of ethical treatment and IACUC of the Second Affiliated Hospital of Soochow University. The protocols of the study were approved by the Ethics Committee (2015-BR021) of the Second Affiliated Hospital of Soochow University.

### Statistical analysis

The investigators were always blinded to the group allocation during the experiments of the study. In vitro experiments were repeated at least three times, with similar results obtained. Data were presented as mean ± standard deviation (SD). Statistics were analyzed by one-way ANOVA followed by a Scheffe’ and Tukey Test (SPSS 19.0, Chicago, IL). A two-tailed unpaired *T* test was applied to test significance between two treatment groups (Excel 2007). Significance was chosen as *p* < 0.05.

## Results

### LIN28B-AS1 is expressed in human HCC cells and tissues

**Fig. 1:**
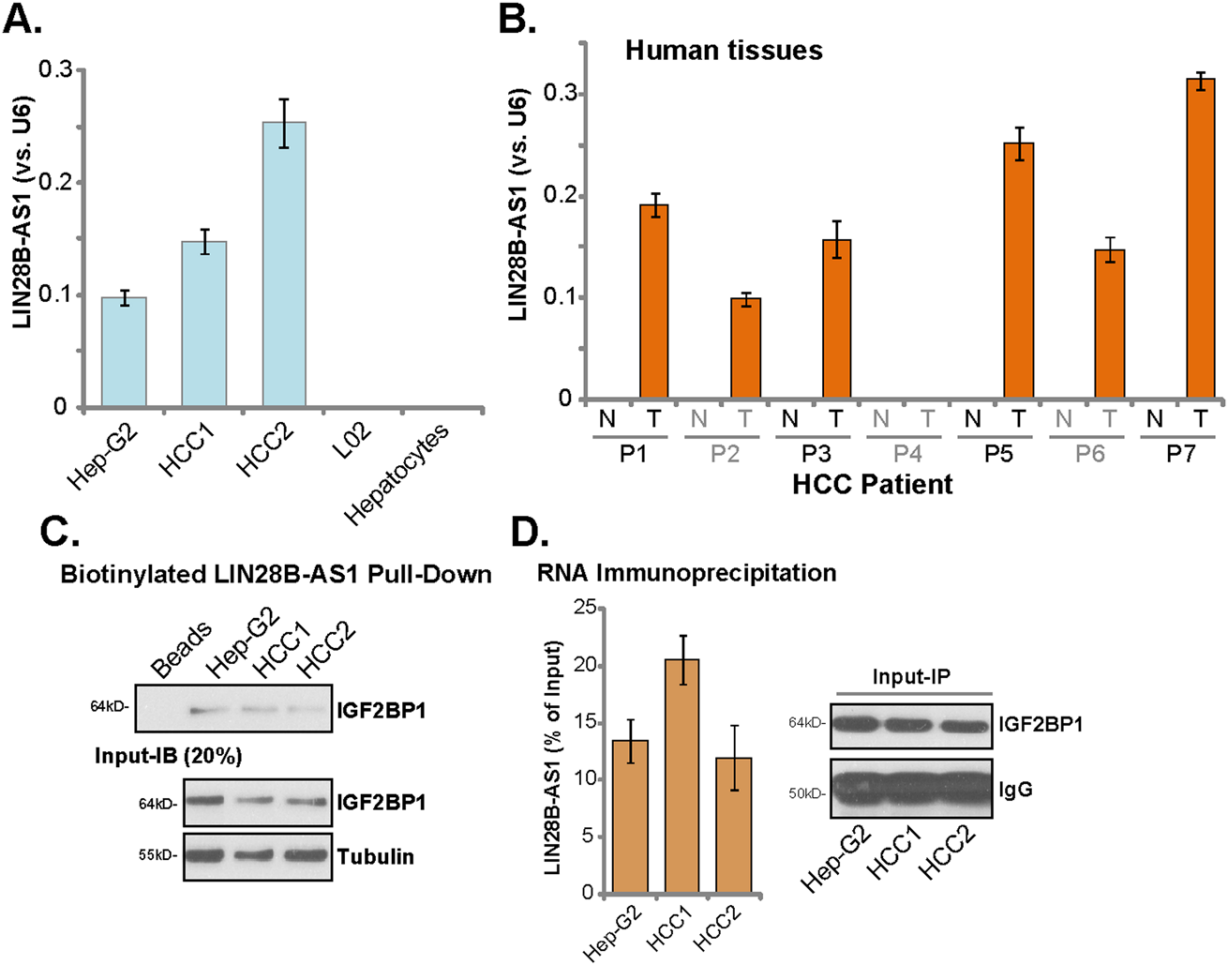
LIN28B-AS1 is expressed in human HCC cells and tissues. Total RNA was extracted from the established/primary human cells, L02 hepatocytes and primary human hepatocytes **a**, and from a total of seven (7) pairs human HCC tissues (“T”) and surrounding normal liver tissues (N”) **b**, LIN28B-AS1 expression was tested by qPCR, and its levels were normalized to U6 **a**, **b**. Western blotting of IGF2BP1 protein retrieved by in vitro-transcribed LIN28B-AS1 in HepG2 and primary human HCC cells **c**. qPCR analyses of LIN28B-AS1 enriched by IGF2BP1 protein in HepG2 and primary human HCC cells **d**. Data were presented as mean ± standard deviation (SD, *n* = 5). The experiments were repeated three times, and similar results were obtained.

First, we tested expression of LIN28B-AS1 in human HCC cells. By employing qPCR, we show that LIN28B-AS1 is expressed in established (HepG2 cell line) and primary human HCC cells (“HCC1/2”) (Fig. 1a). Conversely, its expression is not detected in established L02 hepatocytes and primary human hepatocytes (Fig. 1a). LIN28B-AS1 expression was also detected in six out of seven human HCC tissues (“T”, Fig. 1b). It is however not expressed in all seven normal liver tissues (“N”, Fig. 1b). Thus, LIN28B-AS1 is expressed in human HCC cells and tissues.

To test the possible association between LIN28B-AS1 and the IGF2BP1 protein in HCC cells, the LIN28B-AS1 pull-down assay was carried out. Our results show that the IGF2BP1 protein is precipitated with the in vitro-transcribed and biotinylated LIN28B-AS1 in the nuclei of HepG2 cell and primary human HCC cells (Fig. 1c). Additionally, employing a RIP assay, we again confirmed the direct association between endogenous LIN28B-AS1 and the IGF2BP1 protein in HepG2 cells and primary HCC cells (Fig. 1d). Therefore, LIN28B-AS1 directly associates with the IGF2BP1 protein in HCC cells.

### LIN28B-AS1 silencing inhibits HCC cell progression in vitro

**Fig. 2:**
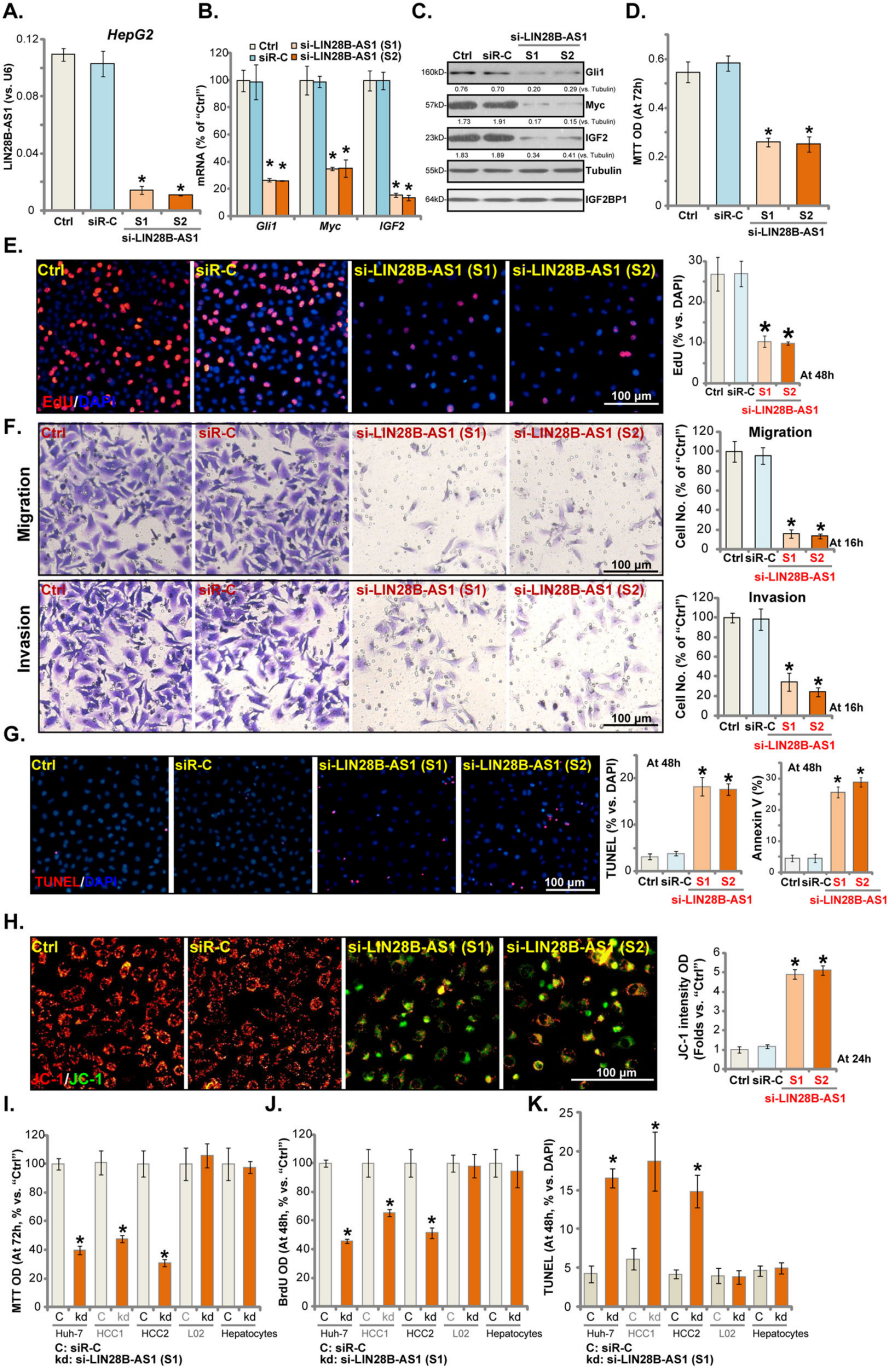
LIN28B-AS1 silencing inhibits HCC cell progression in vitro. HepG2 cells were transfected with LIN28B-AS1 siRNA (“si-LIN28B-AS1-S1/S2”, 0.5 μM) or the non-sense control siRNA (“siR-C”) for 48 h, expression of LIN28B-AS1 **a** and listed genes **b**, **c** was shown; Cells were further cultured for applied time periods, and cell viability was tested by MTT **d**, with cell proliferation examined by EdU staining assay **e**; Cell migration and invasion were tested by “Transwell” and “Matrigel Transwell” assays, respectively **f**. Cell apoptosis and mitochondrial depolarization were examined by TUNEL staining/Annexin V FACS **g** and JC-1 staining **h** assays, respectively. Huh7 cells and primary human HCC cells (“HCC1/2”) as well as L02 or primary human hepatocytes (“Hepatocytes”, same for all figures) were transfected with si-LIN28B-AS1-S1 (“kd”) or non-sense control siRNA (“siR-C”) for 48 h. Cells were further cultured for applied time periods, cell survival and proliferation were, respectively, tested by MTT **i** and BrdU incorporation **j** assays, with cell apoptosis examined by TUNEL staining **k**. For EdU-staining assays, five randomly selected views (of each condition) with total 1000 cells were included to calculate EdU/DAPI ratios (same for all figures). For “Transwell” and “Matrigel Transwell” assays, five randomly selected views in each condition were included to calculate the average number of migrated/invaded cells (same for all figures). For all in vitro functional assays, the exact same number of viable cells of different genetic treatment/s were initially seeded onto each well/dish (“Day-0”/“0 h”, same for all figures). Listed proteins were quantified and normalized **c**. “Ctrl” stands for the parental control cells (same for all figures). Data were presented as mean ± standard deviation (SD, *n* = 5). *p < 0.05 vs. “siR-C” cells. The experiments were repeated three times, and similar results were obtained. Bar = 100 μm.

To study the potential effect of LIN28B-AS1 on HCC cell functions, HepG2 cells were transfected with the LIN28B-AS1 siRNAs (see “Methods” section). The applied siRNA (“S1” or “S2”, with non-overlapping sequences) induced over 90% reduction of LIN28B-AS1 (Fig. 2a). Consequently, IGB2BP1-dependent mRNAs, including *Gli1*, *Myc*, and *IGF2*^17,21,26–28^, were downregulated (Fig. 2b). Gli1, Myc, and IGF2 protein levels were decreased as well (Fig. 2c), while IGF2BP1 protein expression unaffected (Fig. 2c). By performing MTT assay, we show that LIN28B-AS1 silencing by the applied siRNAs inhibited HepG2 cell viability (MTT OD, Fig. 2d). In Fig. 2e, we demonstrated that LIN28B-AS1 siRNA significantly inhibited EdU incorporation in HepG2 cells, indicating proliferation inhibition. Furthermore, HepG2 cell migration and invasion, tested by “Transwell” and “Matrigel Transwell” assays, were suppressed (Fig. 2f). For the “Transwell” assays, cells were incubated for only 16 h to exclude the possible influence of cell migration.

The potential effect of LIN28B-AS1 silencing on HCC cell apoptosis was studied next. As shown in Fig. 2g, in HepG2 cells LIN28B-AS1 siRNA significantly increased the ratio of nuclear TUNEL staining. With LIN28B-AS1 silencing Annexin V ratio was increased in HepG2 cells (Fig. 2g). Furthermore, the JC-1 green monomers accumulation was detected in LIN28B-AS1-silenced HepG2 cells (Fig. 2h), indicating mitochondrial depolarization. These results suggest that LIN28B-AS1 silencing induced apoptosis activation in HepG2 cells. The scramble control siRNA (“siR-C”) did not affect LIN28B-AS1 expression and HepG2 cell functions (Fig. 2a–h).

In Huh-7 cells and primary (“HCC1/2”) human HCC cells, LIN28B-AS1 siRNA (“S1”) inhibited cell viability (Fig. 2i) and BrdU incorporation (Fig. 2j). Yet, the applied LIN28B-AS1 siRNA had no significant effect in L02 hepatocytes and primary human hepatocytes (Fig. 2i, j), where LIN28B-AS1 is not expressed (Fig. 1). Additional apoptosis assays showed that LIN28B-AS1 silencing increased the nuclear TUNEL staining in Huh-7 and primary (“HCC1/2”) HCC cells (Fig. 2k), but being ineffective in L02 cells and primary hepatocytes (Fig. 2k). Thus, LIN28B-AS1 siRNA induced apoptosis activation in the HCC cells. Collectively, these results show that LIN28B-AS1 silencing by targeted siRNA inhibited HCC cell progression in vitro.

### LIN28B-AS1 KO inhibits HCC cell progression in vitro

**Fig. 3:**
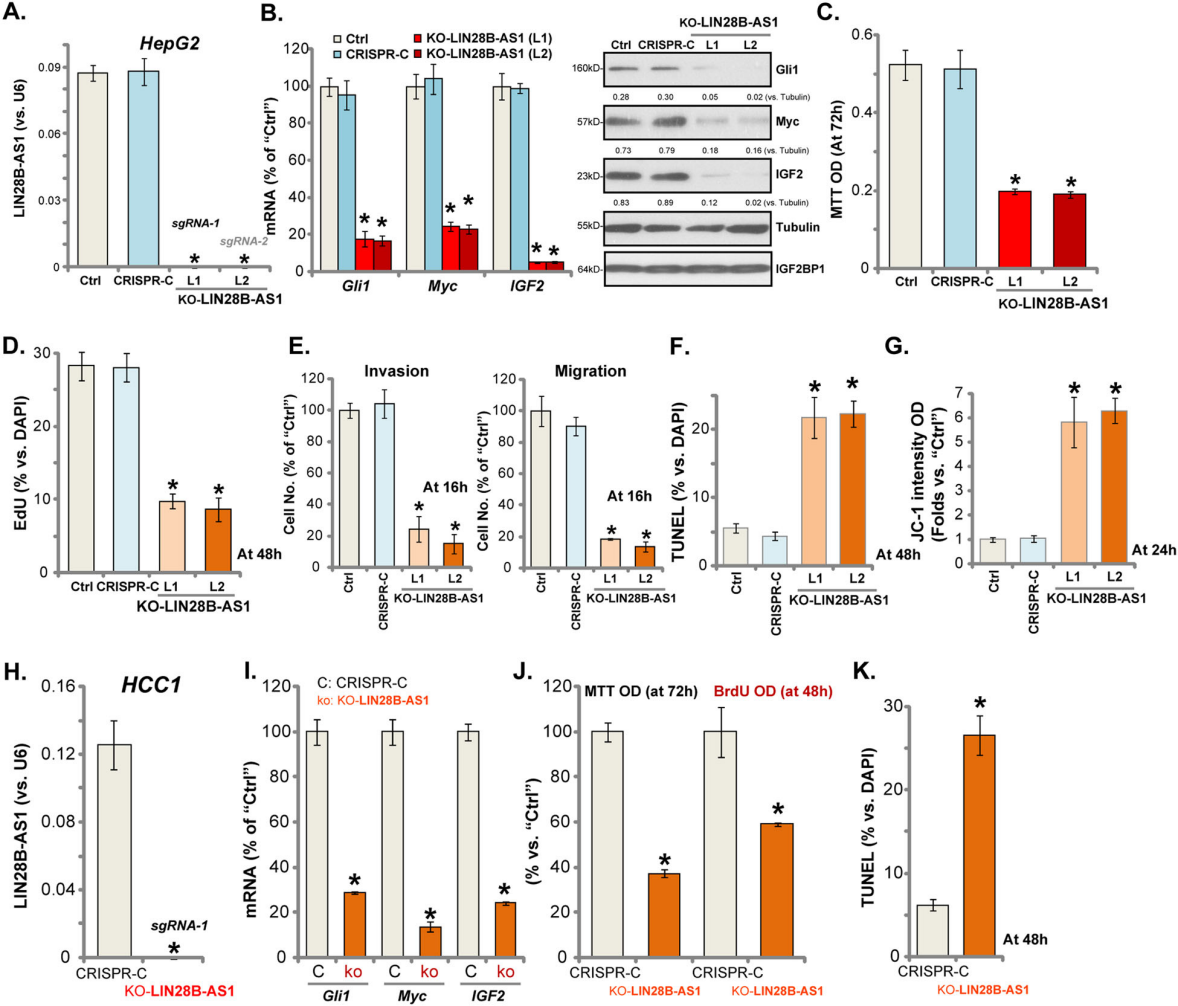
LIN28B-AS1 KO inhibits HCC cell progression in vitro. Total RNA was extracted from the stable HepG2 cells or primary human HCC cells (“HCC1”) with CRISPR/Cas9-LIN28B-AS1-KO construct (“KO-LIN28B-AS1”) or CRISPR/Cas9 control construct (“CRISPR-C”), expression of LIN28B-AS1 **a**, **h**, *Gli1*, *Myc* and *IGF2* mRNA/protein **b**, **i** were tested. Cells were further cultured for applied time periods, cell survival, proliferation, migration, invasion as well as cell apoptosis and mitochondrial depolarization were tested by the assays mentioned, and results were quantified **c**–**g**, **j**, **k**. Listed proteins were quantified and normalized **b**. Data were presented as mean ± standard deviation (SD, *n* = 5). *p < 0.05 vs. “CRISPR-C” cells. The experiments were repeated three times, and similar results were obtained.

To further support a role of LIN28B-AS1 in HCC cell functions, the CRISPR/Cas9-LIN28B-AS1-KO construct (see “Methods” section) was transfected to HepG2 cells. The stable cells were established after GFP sorting by FACS. qPCR results showed that LIN28B-AS1 was completely depleted in the two lines (“L1/L2”) of stable cells with the construct (“KO-LIN28B-AS1”, Fig. 3a). mRNA and protein levels of IGF2BP1-dependent mRNAs, including *Gli1*, *Myc*, and *IGF2*, were dramatically downregulated (Fig. 3b), with IGF2BP1 protein expression unaffected (Fig. 3b). Importantly, LIN28B-AS1 KO potently inhibited HepG2 cell survival, with MTT viability OD decreased (Fig. 3c). EdU/DAPI ratios were decreased as well in LIN28B-AS1-KO HepG2 cells (Fig. 3d). “Transwell” and “Matrigel Transwell” assay results, quantified in Fig. 3e, demonstrated that LIN28B-AS1 KO largely inhibited HepG2 cell migration and invasion in vitro.

Testing cell apoptosis, by TUNEL assay, confirmed that LIN28B-AS1 KO induced significant apoptosis activation (nuclear TUNEL ratio increase) in HepG2 cells (Fig. 3f), simultaneously causing mitochondrial depolarization or JC-1 green monomer accumulation (JC-1 green intensity results quantified in Fig. 3g). Together, these results show that LIN28B-AS1 KO inhibited HepG2 cell progression in vitro.

Using the same CRISPR/Cas9-LIN28B-AS1-KO construct, we completely depleted LIN28B-AS1 in the primary human HCC cells (“HCC1”) (Fig. 3h). *Gli1*, *Myc*, and *IGF2* mRNAs were downregulated in LIN28B-AS1 KO HCC1 cells (Fig. 3i). Cell viability and proliferation were inhibited as well (Fig. 3j). Additionally, LIN28B-AS1 KO augmented positive nuclear TUNEL ratio in HCC1 cells (Fig. 3k), indicating apoptosis activation. Collectively, these results show that LIN28B-AS1 KO inhibited human HCC cell survival and proliferation in vitro.

### Ectopic LIN28B-AS1 overexpression promotes human HCC cell progression in vitro

**Fig. 4:**
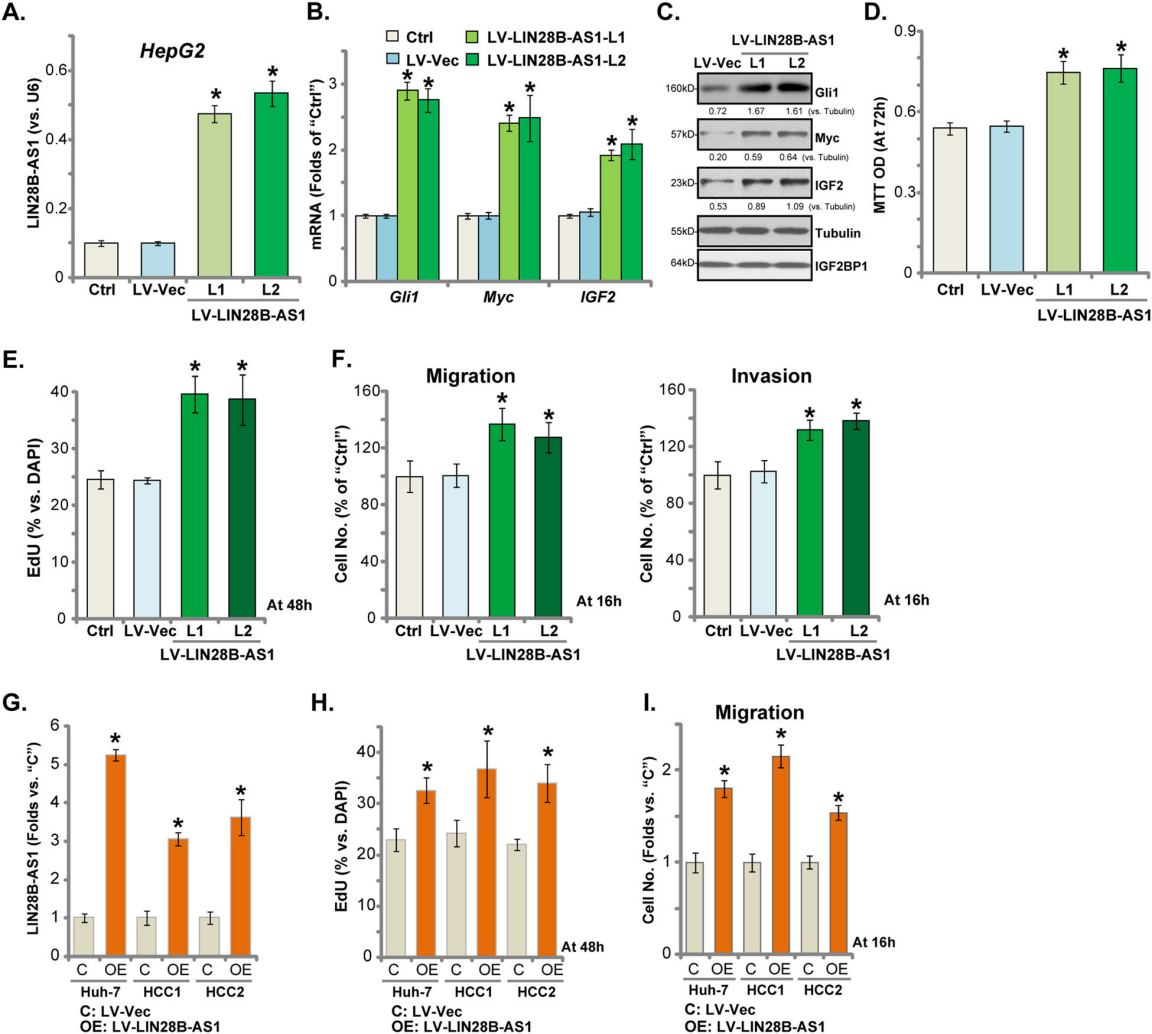
Ectopic LIN28B-AS1 overexpression promotes human HCC cell progression in vitro. Total RNA was extracted from the stable HepG2 cells with the lentiviral pre-LIN28B-AS1 expression construct (“LV-LIN28B-AS1”, two lines, “L1/L2”) or empty vector (“LV-Vec”), LIN28B-AS1 **a** as well as *Gli1*, *Myc*, and *IGF2* mRNAs **b** were tested; Listed proteins were tested by Western blotting **c**. Cells were further cultured for applied time periods, cell survival, and proliferation were tested by MTT **d** and EdU staining **e** assays, respectively; Cell migration and invasion were tested by “Transwell” and “Matrigel Transwell” assays, with results quantified **f**, respectively. Huh7 cells and primary HCC cells (“HCC1/2”) were transduced with LV-LIN28B-AS1 or LV-Vec, and stable cells established with puromycin selection. Expression of LIN28B-AS1 was tested **g**, with cell proliferation and migration examined by EdU incorporation **h** and “Transwell” assays **i**, and results were quantified. Listed proteins were quantified and normalized **c**. Data were presented as mean ± standard deviation (SD, *n* = 5). *p < 0.05 vs. “LV-Vec” cells. The experiments were repeated three times, and similar results were obtained.

Above results using siRNA and CRISPR/Cas9 KO strategies showed that LIN28B-AS1 silencing inhibited HCC cell progression in vitro. Therefore, LIN28B-AS1 overexpression could possibly promote HCC cell progression in vitro. To test this hypothesis, a lentiviral pre-LIN28B-AS1 expression vector (“LV-LIN28B-AS1”) was transduced to HepG2 cells. After puromycin selection stable HepG2 cells (two lines, “L1/L2”) were established, showing over five-folds increase of LIN28B-AS1 expression (Fig. 4a). IGF2BP1’s targets, including *Gli1*, *Myc*, and *IGF2*, were significantly increased in LIN28B-AS1-overexpressed cells (Fig. 4b, c), with IGF2BP1 protein expression unchanged (Fig. 4c). Importantly, HepG2 cell viability (MTT OD, Fig. 4d) and proliferation (EdU-positive nuclei ratio, Fig. 4e) were augmented with LIN28B-AS1 overexpression. Furthermore, HepG2 cell migration and invasion, tested by “Transwell” and “Matrigel Transwell” assays, respectively, were enhanced in LIN28B-AS1-overexpressed HepG2 cells (results quantified in Fig. 4f).

In Huh-7 cells and primary (“HCC1/2”) human HCC cells, LV-LIN28B-AS1 similarly increased LIN28B-AS1 overexpression (3–6 folds of control level) (Fig. 4g). Exogenous LIN28B-AS1 overexpression promoted HCC cell proliferation (EdU-positive nuclei ratio, Fig. 4h) and migration (“Transwell” assay, results quantified in Fig. 4i). These results further supported an essential role of LIN28B-AS1 in regulating HCC cell functions.

### Ectopic IGF2BP1 overexpression is ineffective on the functions of LIN28B-AS1 KO HepG2 cells

**Fig. 5:**
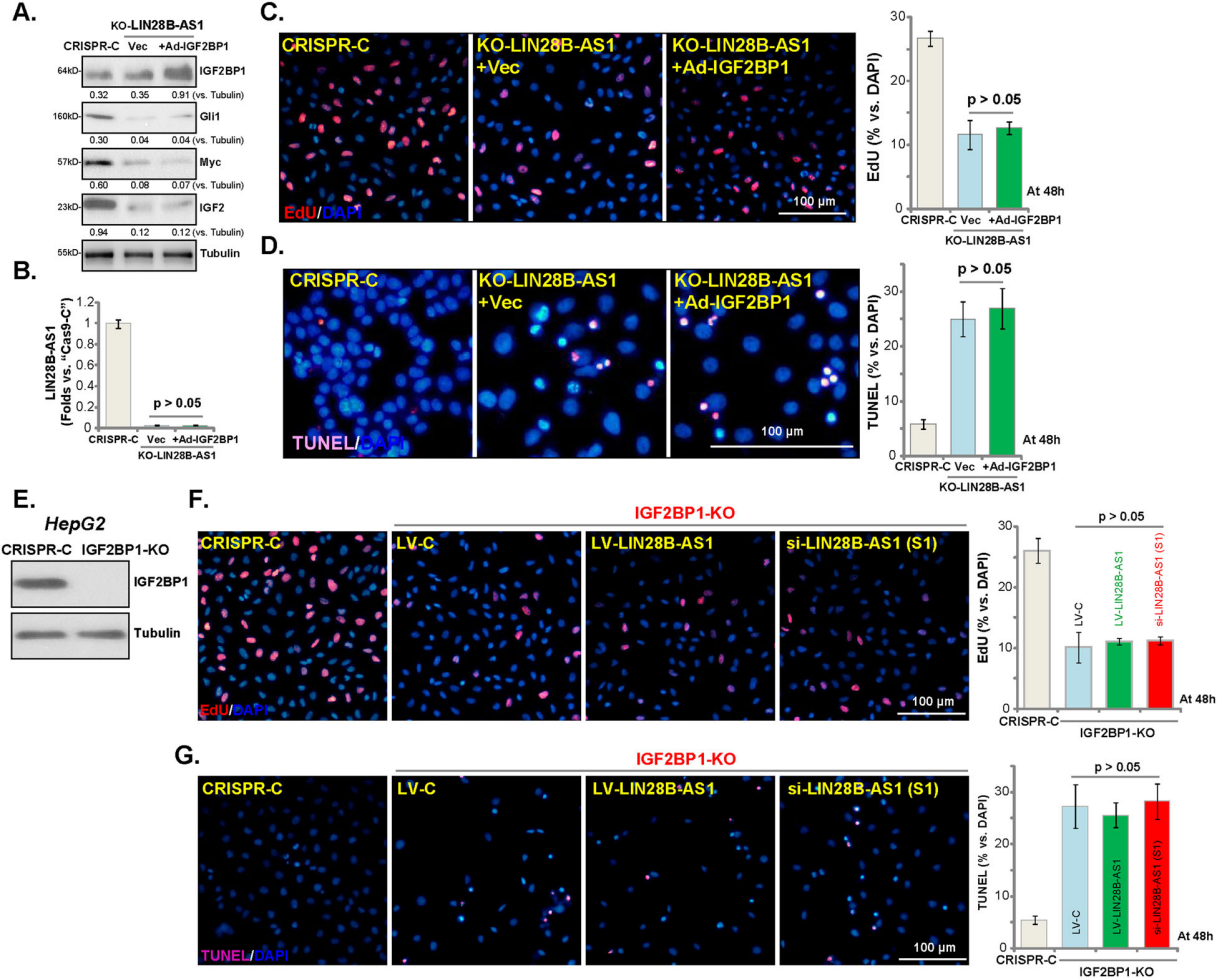
Ectopic IGF2BP1 overexpression is ineffective on the functions of LIN28B-AS1 KO HepG2 cells. The stable HepG2 cells with CRISPR/Cas9-LIN28B-AS1-KO construct (“KO-LIN28B-AS1”) were further infected with recombinant adenovirus encoding the human IGF2BP1 expression construct (“Ad-IGF2BP1”) or empty vector (“Vec”), control cells were transduced with the CRISPR/Cas9 control construct (“CRISPR-C”); Expression of listed proteins **a** and LIN28B-AS1 **b** was shown; Cells were further cultured for 48 h, cell proliferation and apoptosis were tested by EdU incorporation **c** and TUNEL staining **d** assays, respectively. Expression of IGF2BP1 and Tubulin in stable HepG2 cells with the CRISPR/Cas9-IGF2BP1-KO construct (IGF2BP1-KO cells) or control cells (“Ctrl”) was shown **e**; IGF2BP1-KO cells were further transfected with LIN28B-AS1 siRNA (“si-LIN28B-AS1-S1”, 0.5 μM) or LV-LIN28B-AS1 for 48 h, cell proliferation **f** and apoptosis **g** were tested similarly. Listed proteins were quantified and normalized **a**. Data were presented as mean ± standard deviation (SD, *n* = 5). The experiments were repeated three times, and similar results were obtained. Bar = 100 μm.

Next, tested whether exogenous IGF2BP1 overexpression could rescue the LIN28B-AS1 KO HCC cells. The IGF2BP1-expressing recombinant adenovirus, Ad-IGF2BP1 (from Dr. Liu^24^), was transfected to LIN28B-AS1-KO HepG2 cells (“L1”, see Fig. 3), resulting in IGF2BP1 overexpression within 48 h (Fig. 5a). However, Ad-IGF2BP1 failed to affect the decreased expression of Gli1, Myc, and IGF2 in LIN28B-AS1 KO cells (Fig. 5a). It certainly did not change LIN28B-AS1 expression (Fig. 5b). Functional studies showed that LIN28B-AS1 KO-induced proliferation inhibition (EdU incorporation, Fig. 5c) and apoptosis (TUNEL staining, Fig. 5d) were not attenuated by ectopic IGF2BP1 overexpression. These results show that ectopic IGF2BP1 overexpression failed to rescue the LIN28B-AS1 KO HepG2 cells, suggesting that LIN28B-AS1 is essential for IGF2BP1’s functions.

If LIN28B-AS1-induced HCC cell progression is via association with IGF2BP1, LIN28B-AS1 should be ineffective in IGF2BP1 KO cells. To test this hypothesis, the CRISPR/Cas9-IGF2BP1-KO construct (from Dr. Liu^24^) was transduced to establish IGF2BP1-KO HepG2 cells, where IGF2BP1 is completely depleted (Fig. 5e). As expected, IGF2BP1 KO inhibited HepG2 cell proliferation (EdU incorporation, Fig. 5f), while inducing apoptosis activation (TUNEL staining increase, Fig. 5g). Importantly, LIN28B-AS1 siRNA (“S1”, see Fig. 2) or LIN28B-AS1 overexpression (by LV-LIN28B-AS1, see Fig. 4) failed to significantly alter proliferation and apoptosis in IGF2BP1 KO HepG2 cells (Fig. 5f, g). These results confirm our hypothesis that LIN28B-AS1 promotes HCC cell progression by binding to IGF2BP1.

### LIN28B-AS1 KO inhibits HepG2 xenograft tumor growth in mice

**Fig. 6:**
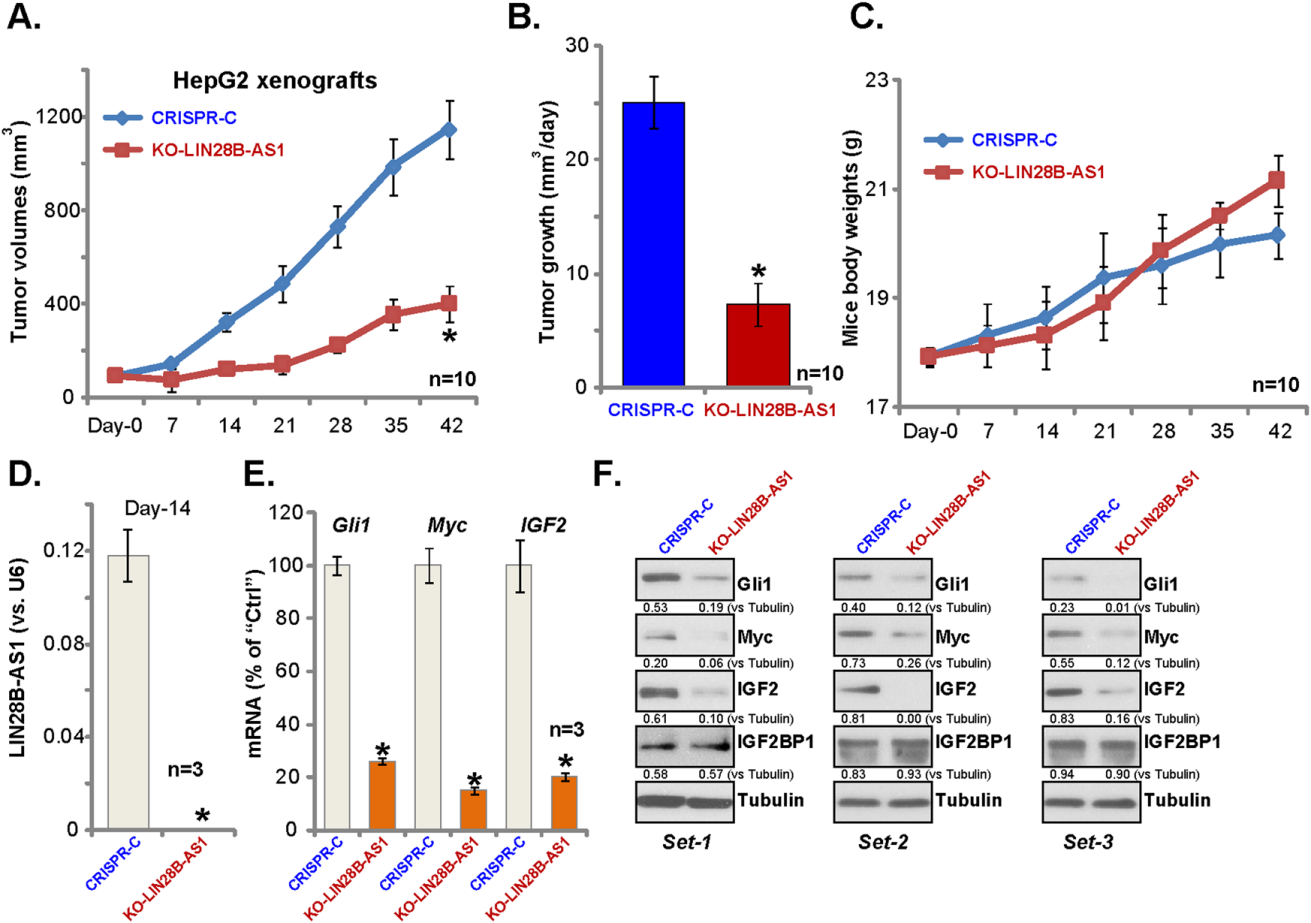
LIN28B-AS1 KO inhibits HepG2 xenograft tumor growth in mice. LIN28B-AS1 KO (“KO-LIN28B-AS1”) or the control (“CRISPR-C”) HepG2 cells were inoculated s.c. to the flanks of the nude mice; Tumor volumes **a** and mice body weights **c** were recorded every 7 days for a total of 42 days. Daily tumor growth was calculated as described **b**; At Day-14, three HepG2 tumors of each group were isolated, expression of LIN28B-AS1 and listed genes were shown **d**–**f**. Bars stand for mean ± standard deviation (SD). *p < 0.05 vs. “CRISPR-C” tumors.

At last, we tested the potential effect of LIN28B-AS1 on HCC cell growth in vivo. Control HepG2 cells and LIN28B-AS1 KO HepG2 cells (“L1”, see Fig. 3) were inoculated s.c. to the flanks of the nude mice. Recordings were started when tumor volumes were close to 100 mm^3^ (“Day-0”). We show that LIN28B-AS1 KO HepG2 xenografts grew significantly slower than control tumors (Fig. 6a). The estimated daily tumor growth (in mm^3^ per day) was also calculated: (volume at Day-42 subtracting volume at Day-0)/42. The results further confirmed that LIN28B-AS1 KO potently inhibited HepG2 tumor growth (Fig. 6b). The mice body weights were not significantly different between two groups (Fig. 6c).

At Day-14, three tumors of each group were isolated, and the tumor tissue lysates were obtained. Expectably, LIN28B-AS1 was depleted in LIN28B-AS1 KO tumor tissues (Fig. 6d), where IGB2BP1-dependent mRNAs, including *Gli1*, *Myc*, and *IGF2*, were decreased (Fig. 6e). Protein levels of Gli1, Myc, and IGF2 were significantly downregulated as well in LIN28B-AS1 KO tumor tissues (Fig. 6f), with IGF2BP1 protein levels unchanged (Fig. 6f). Therefore, LIN28B-AS1 KO inhibits HepG2 xenograft tumor growth in mice.

## Discussion

LncRNAs are a large class of transcripts with largely unknown biological functions. Existing studies have implied that LncRNA dysregulation could play a pivotal role in the initiation, tumorigenesis, and progression of HCC^10–13^. LncRNAs can crosstalk with multiple chromatin, DNA, RNA, and proteins, regulating HCC cell progression via transcriptional and post-transcriptional mechanisms^10–13^. Hosono et al. in 2017 has first identified a conserved cancer/testis Lnc-RNA, namely THOR (Lnc-THOR). It promoted cancer progression through directly interacting with IGF2BP1^5^. Very recent studies have shown that Lnc-THOR-IGF2BP1 association is important for HCC cell proliferation and migration, as well as liver cancer stem cells expansion^29,30^. Inhibition or disruption Lnc-THOR-IGF2BP1 association potently inhibited human cancer cell progression^29,30^. These results highlight that IGF2BP1-bound LncRNAs should be novel and important therapeutic targets for HCC^29,30^.

IGF2BP1 is one key member of the RNA-binding IGF2BP family proteins. It is essential for mRNA stabilization and translation of several key pro-cancerous genes. Our results confirmed that LIN28B-AS1-IGF2BP1 association is essential for IGF2BP1 activity. We show that LIN28B-AS1 is expressed in human HCC cells and tissues. It is however not expressed in human hepatocytes and normal liver tissues. RIP and RNA pull-down assay results confirmed the direct association between LIN28B-AS1 and the IGF2BP1 protein in HCC cells.

Significantly, LIN28B-AS1 siRNA or KO downregulated IGF2BP1-dependent mRNAs (*IGF2*, *Gli1*, and *Myc*), leading to potent inhibition on HCC cell growth, proliferation, migration, and invasion. Conversely, forced overexpression of LIN28B-AS1, by a lentiviral construct, promoted HCC cell progression in vitro. In vivo, LIN28B-AS1 KO-HepG2 tumors grew significantly slower than the control tumors in the nude mice. These results indicate that LIN28B-AS1-IGF2BP1 binding is essential for IGF2BP1’s functions in HCC cells.

The fact that neither LIN28B-AS1 siRNA nor LIN28B-AS1 overexpression was effective in IGF2BP1-KO HCC cells indicates that LIN28B-AS1-mediated HCC cell progression requires binding to IGF2BP1. The further studies demonstrated that ectopic overexpression of IGF2BP1, by Ad-IGF2BP1, failed to rescue LIN28B-AS1-KO HepG2 cells.

## Conclusion

These results together suggest that LIN28B-AS1 associates with IGF2BP1 to promote human HCC cell progression in vitro and in vivo. LIN28B-AS1 could be a novel and valuable therapeutic target for HCC.
